# Intracellular Uptake: A Possible Mechanism for Silver Engineered Nanoparticle Toxicity to a Freshwater Alga *Ochromonas danica*

**DOI:** 10.1371/journal.pone.0015196

**Published:** 2010-12-22

**Authors:** Ai-Jun Miao, Zhiping Luo, Chi-Shuo Chen, Wei-Chun Chin, Peter H. Santschi, Antonietta Quigg

**Affiliations:** 1 State Key Laboratory of Pollution Control and Resource Reuse, School of Environment, Nanjing University, Nanjing, Jiangsu Province, People's Republic of China; 2 Department of Marine Science, Texas A & M University at Galveston, Galveston, Texas, United States of America; 3 Department of Marine Biology, Texas A & M University at Galveston, Galveston, Texas, United States of America; 4 Microscopy and Imaging Center, Texas A & M University, College Station, Texas, United States of America; 5 School of Engineering, University of California Merced, Merced, California, United States of America; 6 Department of Oceanography, Texas A & M University, College Station, Texas, United States of America; University of Kansas, United States of America

## Abstract

The behavior and toxicity of silver engineered nanoparticles (Ag-ENs) to the mixotrophic freshwater alga *Ochromonas danica* were examined in the present study to determine whether any other mechanisms are involved in their algal toxicity besides Ag^+^ liberation outside the cells. Despite their good dispersability, the Ag-ENs were found to continuously aggregate and dissolve rapidly. When the initial nanoparticle concentration was lower than 10 µM, the total dissolved Ag^+^ concentration ([Ag^+^]_T_) in the suspending media reached its maximum after 1 d and then decreased suggesting that Ag^+^ release might be limited by the nanoparticle surface area under these conditions. Furthermore, Ag-EN dissolution extent remarkably increased in the presence of glutathione. In the Ag-EN toxicity experiment, glutathione was also used to eliminate the indirect effects of Ag^+^ that was released. However, remarkable toxicity was still observed although the free Ag^+^ concentration in the media was orders of magnitude lower than the non-observed effect concentration of Ag^+^ itself. Such inhibitive effects were mitigated when more glutathione was added, but could never be completely eliminated. Most importantly, we demonstrate, for the first time, that Ag-ENs can be taken in and accumulated inside the algal cells, where they exerted their toxic effects. Therefore, nanoparticle internalization may be an alternative pathway through which algal growth can be influenced.

## Introduction

Engineered nanoparticles of silver (Ag-ENs), being one of the most important ENs, are now extensively used as bactericides or fungicides and have found versatile applications in diverse products like household appliances, cleaners, clothing, cutlery, children's toys, and coated electronics [1]. They are believed to be the most commercialized nanomaterial [2]. As a result of their wide applications, a considerable fraction of the Ag-ENs will eventually find their way into aquatic ecosystems and possibly exert some negative effects, given their anti-bacterial characteristics. With concerns of potential eco-risks, a coalition of consumer, health, and environmental groups filed a legal petition with the U.S. Environmental Protection Agency, demanding that the agency use its authority to regulate pesticides to stop the sale of more than 260 consumer products containing nanosized versions of silver in 2008 [3].

Holding the same concerns, the potential toxicity of Ag-ENs together with their underlying toxicity mechanisms has been extensively investigated [1], [4], [5], [6]. It has been widely accepted that Ag-EN dissolution (i.e., the release of Ag^+^) plays an important role in their toxicity to different organisms. However, to what extent dissolution is involved is still in debate. For example, Navarro et al. [7] found that the interaction between the carbonate-coated Ag-ENs and the freshwater alga *Chlamydomonas reinhardtii* influences the nanoparticle toxicity, which is mediated by Ag^+^, and thus concluded that Ag-ENs are toxic by serving as a source of Ag^+^. On the other hand, there are also studies in which Ag-EN toxicity could not be completely explained by their liberation of Ag^+^ [8], [9], . Griffitt et al. [8] tested the toxicity of silver, copper, aluminum, nickel, and cobalt as both nanoparticles and soluble salts to zebrafish, daphnids, and an algal species. They found that the role of dissolution in observed toxicity varied, being minor for silver and copper but, apparently, accounting for most of the toxicity of nickel. Carlson et al. [11] examined the toxicity of different nano-sized Ag-ENs (15, 30, 55 nm) to alveolar macrophages. The nanoparticle toxicity was found to increase with decreasing size. More interestingly, there was a remarkable uptake of Ag-ENs by macrophages as was directly visualized by both transmission electron microscope (TEM) and inverted light microscopy. Therefore, it appears that the toxicity mechanisms of Ag-ENs are associated with their chemical composition and is organisms or cell species specific.

In our previous study, a series of Ag-EN algal toxicity experiments were also carried out in seawater system to investigate how bare Ag-ENs might be harmful to a coastal marine diatom *Thalassiosira weissflogii* [12]. Ag-ENs were found to aggregate rapidly in seawater, whereby a considerable amount of Ag^+^ was released during a 24 h period, which was found to be the main cause for the observed toxicity. Accordingly the inhibitive effects from Ag-ENs disappeared completely when either 0.45 mM glutathione (GSH) or 1.10 mM cysteine was added even though the presence of these thiols could induce the dispersion of Ag-ENs into the <0.22 µm fraction. It was thus proposed that other toxicity pathways than Ag^+^ liberation outside the algal cells for Ag-ENs might only be observed with smaller particle sizes (<60–70 nm), different surface coatings, or at nanoparticle concentrations >10 µM.

To further test the above hypothesis that other mechanisms exist for Ag-EN toxicity to phytoplankton, surface functionalized hydrophilic Ag-ENs were chosen in the present study. A freshwater system was used to ensure most of Ag-ENs were distributed in the <100 nm fraction instead of rapidly aggregated as seen in seawater. Furthermore, a mixotrophic freshwater alga *Ochromonas danica* (Chrysophyceae) was adopted, which is well known for its ability to take in bacteria as an additional carbon source through phagocytosis [13]. Therefore, this alga might also be able to take up Ag-ENs in a similar way. If Ag-ENs could affect phytoplankton through some other way, we hypothesize it should be more likely to be observed in a mixotrophic species. To the best of our knowledge, there are no currently published reports on the internalization of nanoparticles into the algal cells. A series of experiments on the behavior of Ag-ENs were also performed before the toxicity tests in order to examine how Ag-ENs were distributed in the different size fractions and how much Ag^+^ was released under different conditions during the toxicity experiments.

## Materials and Methods

### Phytoplankton culture conditions

The chrysophyte *Ochromonas danica* (UTEX 1298) used in this study was obtained from the Provasoli-Guillar

d Center for the Culture of Marine Phytoplankton, Bigelow Laboratory, West Boothbay Harbor, ME, USA. The algal cells were maintained in a modified DY-V medium (Table S1) at a pH value 6.8±0.1 [14]. As this alga is mixotrophic, glucose and yeast extract were included as additional carbon sources. The pH of the culture media was kept constant with 2 mM MOPS (3-(N-morpholino)-propanesulfonic acid). The temperature was 20°C with a light illumination of 170 µmol photons/m^2^/s in a 12∶12 Light-Dark cycle.

### Ag-EN behavior experiments

The stock solution of the carboxy-functionalized Ag-ENs (1.5 mg/ml, pH 5–7) used in the present study was purchased from Vivo Nano (Toronto, Canada) and was well dispersed in water. More than 90% of the particles were in the 1–10 nm range and more than 97% of the metal content in the solution was accounted for by Ag as reported by the manufacturer. Whenever ‘Ag’ is used in the present paper, it includes both Ag^+^ and Ag-ENs unless specified as Ag^+^ or Ag-ENs. After receiving this product, TEM (FEI Tecnai G^2^ F20 with a field emission gun at a working voltage of 200 kV) was used to confirm the Ag-EN size distribution following a similar procedure to that published previously [12].

Ag-EN behavior experiments were conducted to examine how different parameters (i.e., the mixing time, nanoparticle and GSH concentration) related to the toxicity tests described below affect the Ag-EN size distribution and dissolution. To investigate the effects of mixing time, 27.8 µM (Ag element based molarity) Ag-ENs were added into DY-V medium (in triplicates and 100 ml each) and kept stirring in acid cleaned Teflon beakers for 20 d during which time three aliquots (0.5 ml each) were taken from each triplicate and centrifuge-filtered through a 10 kilodalton (kD), 300 kD, and 0.20 µm membrane, respectively, on day 0, 2, 4, 7, 10, 15 and 20. The 10 and 300 kD ultracentrifugal devices were bought from PALL (Nanosep series) while the 0.20 µm centrifugal filters were obtained from Millipore. After that, the filtrates were digested in 1 ml ultrapure HNO_3_ concentrate and their Ag concentrations measured with a Perkin-Elmer 5100 graphite furnace atomic absorption spectrophotometer (Perkin-Elmer, Wilton, CT, USA). A good mass balance was always obained in the preliminary experiments as well as our previous studies [12]. Therefore, the amount of Ag retained on the filter membrane was not measured in the formal experiment. Blank filters and the potential Ag^+^ retention by the membranes were both quantified to ensure the amount of Ag^+^ from the blank filter or that intercepted by the membrane is negligible. Yeast extract, as a mixture of proteins, amino acids and carbohydrates etc., was excluded from the DY-V media used in both the behavior and toxicity experiments to simplify the whole system. The pore size is around 1 nm and 35 nm for the 10 kD and 300 kD membranes, respectively, as reported by the manufacturer. Therefore, Ag distribution in the <1 nm (truly dissolved Ag^+^), 1 nm–35 nm (primary Ag-ENs and small Ag-EN aggregates, defined as Ag concentration in the <35 nm filtrate minus that in the <1 nm fraction), 35 nm–200 nm (bigger Ag-EN aggregates, defined as Ag concentration in the <200 nm filtrate minus that in the <35 nm fraction), and >200 nm (Ag-EN particulates, defined as the total Ag concentration at the beginning minus that in the <200 nm fraction) fractions could be operationally defined this way. The pH of each triplicate was monitored throughout the whole experiment. The TEM images of Ag-ENs in the <200 nm fraction of each triplicate were then taken to have a better view of their morphology and size distribution at the end of this experiment. The light absorbance (wavelength: 300–800 nm) of Ag-ENs in this fraction was also investigated through a UV Visible spectrophotometer (Shimadzu) both at the beginning and end of this experiment as an alternative method to examine the potential aggregation or dissolution of Ag-ENs.

The other Ag-EN behavior experiments were similarly performed. However, the experimental duration was shortened to 7 d with five time points (day 0, 1, 2, 4, 7) based on the results of the 20 d experiment described above. In the nanoparticle concentration effect experiment, four different Ag-EN concentrations (1.85, 9.27, 27.8, 92.7 µM) were used. As the cysteine containing tripeptide GSH was used to eliminate the effects of Ag^+^ in the toxicity experiments below, the effects of different GSH concentrations (i.e., 0, 16.7, 83.3, and 416.3 µM) were examined to see how GSH may affect the Ag-EN size distribution and dissolution. The behavior experiments above provided valuable information for the toxicity experiment design and the data interpretation of these toxicity tests.

### Ag-EN toxicity experiments

In order to see whether Ag-ENs have any other toxic effects on *O. danica* besides those from Ag^+^, two different toxicity tests were performed using similar media as those in the behavior experiments above (i.e., the base DY-V medium excluding the yeast extract). In the first experiment, the toxicity of Ag-ENs was compared with that of Ag^+^. There were eleven treatments in triplicates (150 ml each) for this experiment. Five of them were spiked with different concentrations of Ag-ENs (27.8, 92.7, 139.1, 185.4, and 278.1 µM) and another five with different Ag^+^ (55.4, 74.2, 81.9, 83.4, and 92.7 µM, nominal concentrations of Ag^+^ initially added). Such concentrations of Ag-EN and Ag^+^ were chosen to ensure the observation of complete dose-response curves based on the results of the preliminary experiments. The eleventh served as a control without any addition of Ag-ENs or Ag^+^. Glutathione (83.3 µM) was used to keep the free Ag^+^ concentration ([Ag^+^]_F_) constant during the experimental period and to eliminate the effects of Ag^+^ in the Ag-EN addition treatments as well. The pH of the toxicity media was kept at 6.8±0.1 throughout the experiment by the addition of 2 mM MOPS. All the media of the different treatments were made one day in advance and left overnight to equilibrate. Right before the start of the toxicity experiment, 0.5 ml aliquots in triplicates from each treatment were filtered through a 10 kD membrane and the total dissolved concentrations of Ag^+^ ([Ag^+^]_T_) in the filtrates were measured. Based on these results, [Ag^+^]_F_ was then calculated using the MINEQL+ software package (Version 4.5 from Environmental Research Software, Hallowell, ME, USA) with updated thermodynamic constants and calibrated ionic strength data.

The algae to be used in the toxicity test were first acclimated under the same conditions as the following experiment (temperature: 20°C, light intensity: 170 µmol photons/m^2^/s, light-dark cycle: 12∶12), except that no Ag-ENs or Ag^+^ were added. Yeast extract was supplied during this period to obtain enough cell biomass. After arriving at the mid-exponential growth phase, the cells were collected, washed and resuspended into the toxicity media prepared as above. The initial cell density, as was enumerated under a compound microscope, ranged from 9.06×10^4^ to 1.42×10^5^ cells/ml for the different treatments. This experiment lasted for two days during which the algal cell density was measured every day and the average cell specific growth rate µ was calculated [12]. The size distribution of Ag-ENs at the end of the toxicity test was also examined by ultrafiltration similar to the behavior experiments above. Further, the algal cells from certain treatments of this experiment were collected to directly visualize whether Ag-ENs can enter the cells with TEM and Z-contrast dark-field scanning transmission electron microscope (STEM) following a similar procedure described by Han et al. [15]. A detailed description about how the samples were prepared is included in Text S1.

The procedure of the second toxicity test was the same as the first one except that Ag-EN toxicity was examined under different concentrations of GSH in this experiment. As the sulphurhydryl group of GSH is a strong Ag^+^ binding ligand, any toxicity of Ag^+^ from Ag-EN dissolution was expected to be completely eliminated when GSH concentration was high enough (i.e., [GSH] ≫ [Ag^+^]_T_). For this purpose, three different concentrations of GSH, 83.3, 249.8, and 416.3 µM, were used and there were four different Ag-EN concentration treatments (i.e., 0, 139.1, 185.4, and 278.1 µM Ag-ENs) for each GSH concentration group resulting in a total of twelve treatments. A brief summary of the experimental design for the three behavior and two toxicity experiments above is shown in Table S2.

### Statistical analysis

Median (50%) effect concentrations (EC50) of free Ag^+^ in the different toxicity tests were calculated using the linear interpolation method (ICPIN software, Version 2.0, USEPA, Duluth, MN, USA). Any ‘significant’ difference in this study was based on results of one-way or two-way analysis of variance with post-hoc multiple comparisons (Turkey or Tamhane) (SPSS 11.0 by SPSS, Chicago, USA). Significant differences were accepted at p<0.05. The normality (Kolmogorov–Smirnov and Shapiro–Wilk tests) and homogeneity of variance (Levene's test) of the data were both examined when performing the analysis of variance.

## Results and Discussion

### Variation of Ag-EN size distribution and their dissolution with mixing time

**Figure 1:**
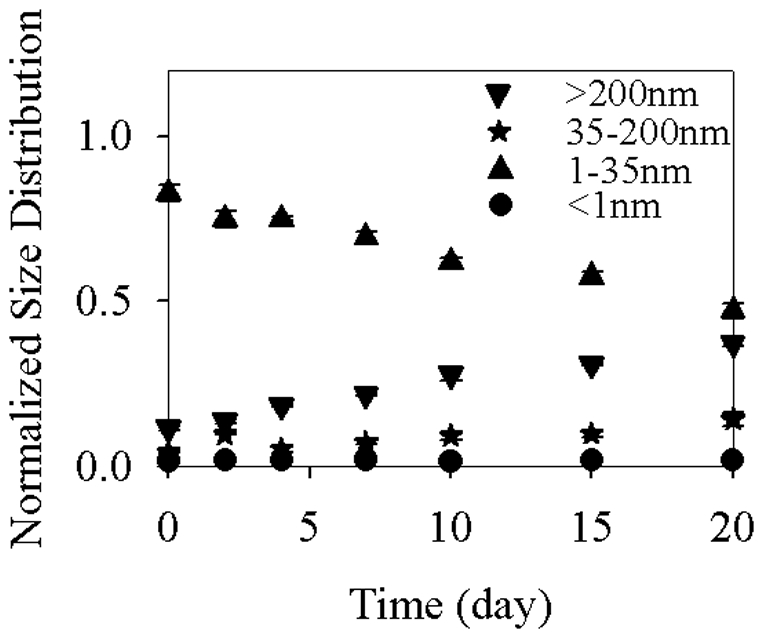
Normalized size distribution (to the initial Ag-EN concentration) of 27.8 µM Ag-ENs in different size fractions (> 200 nm, 35–200 nm, 1–35 nm, <1 nm) during a 20 d period in the modified DY-V medium. Data are mean ± standard deviation (n = 3).

The Ag-ENs used in the present study were well dispersed in both the stock solution and the modified DY-V medium, as evidenced by their TEM images shown in Fig. S1 and by their characteristic light absorption peak at 420 nm in Fig. S2a. The light absorption spectrum of Ag-ENs obtained in the present study was similar to that in other studies [16] and also similar to what was provided by the manufacturer. The normalized size distribution was calculated as the Ag mass in each size range divided by the total amount of Ag-ENs initially added. Most of the nanoparticles (i.e., 82.6%) were found in the 1–35 nm fraction right after their addition to the media of the 20 d experiment (Fig. 1). However, while the Ag-ENs were surface coated with the hydrophilic ligands (polyacrylate sodium), they kept aggregating during the experimental period. Accordingly, the Ag concentration in the 1–35 nm fraction decreased from 23.0 µM on day 0 to 13.1 µM on day 20 while continuously increasing from 1.18 to 3.95 µM and from 3.20 to 10.3 µM in the 35–200 nm and >200 nm fractions, respectively. As a result of their aggregation, the light absorbance by Ag-ENs at the end of this experiment was lower than that at the beginning (Fig. S2a).

As for Ag-EN dissolution, it took place rather quickly, with [Ag^+^]_T_ reaching a maximum concentration in approximately 1 h (the time lag between the start of this experiment and the first sampling for ultrafiltration) despite the fact that surface coating was expected to slow down the dissolution of nanoparticles [17], [18]. The maximum [Ag^+^]_T_ (0.553 µM) observed in the present study was consistent with what was calculated (0.678 µM) by the MINEQL+ software for a medium saturated with Ag bulk particles, suggesting that [Ag^+^]_T_ was limited by Ag-EN solubility in the media. Therefore, the much lower [Ag^+^]_T_ observed in the present study (0.395–0.553 µM) than that in our previous experiment (22.1 µM) with bare Ag-ENs [12] was mainly caused by the nanoparticle solubility difference between a DY-V freshwater medium and artificial seawater, with the latter having an extremely high concentration of chloride (0.56 M), which acts as the major Ag^+^ binding ligand. Furthermore, the quick arrival of a maximum in [Ag^+^]_T_ in this 20 d experiment was possibly due to the combined effects from both the Ag^+^ binding ligands in the DY-V media as well as the ultra-small particle size in the nano range, considering the remarkable influence of solution ligands and surface area on particle solubilization [17].

### Effects of nanoparticle concentration

**Figure 2:**
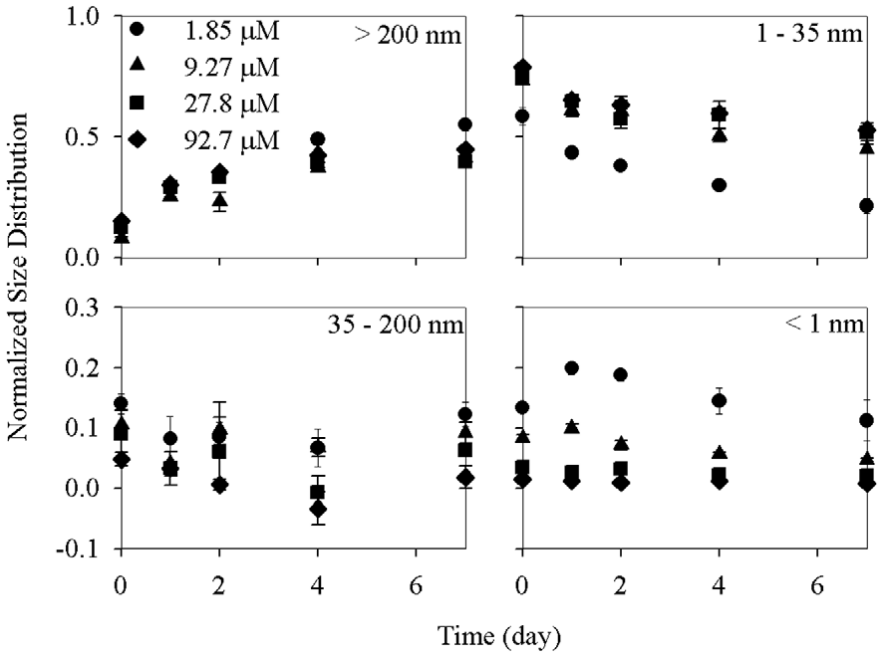
Normalized size distribution (to the initial Ag-EN concentration) of 1.85, 9.27, 27.8, and 92.7 µM Ag-ENs in different size fractions (> 200 nm, 35–200 nm, 1–35 nm, <1 nm) during a 7 d period in the modified DY-V medium. Data are mean ± standard deviation (n = 3).

Similar to the 20 d experiment, the normalized Ag-EN distribution in the >200 nm fraction increased while less Ag-ENs were left in the 1–35 nm fraction with time for the different nanoparticle concentration treatments (Fig. 2). However, the Ag-EN distribution in the 35–200 nm fraction was kept constant during the 7 d period as compared with that in the 20 d experiment suggesting that a considerable change in nanoparticle distribution into this fraction may only be observed after 7 d. Ag distribution in 35 nm–200 nm fraction was obtained by subtracting the Ag concentration in the <35 nm fraction from that in the <200 nm filtrate. Therefore, the negative amount of Ag observed in the 35–200 nm fraction doesn't really mean there are negative amounts of Ag in these fractions but indicates the amount of Ag distributed in these fractions could be neglected, as they are within the error of the determination [19]. Although the Ag-EN distribution in the <1 nm (10 kD) fraction remained unchanged with time in the two highest nanoparticle concentration treatments (27.8 and 92.7 µM), [Ag^+^]_T_ increased from 0.25 and 0.77 µM at the beginning to 0.37 and 0.92 µM on day 1 and then decreased continuously to 0.21 and 0.43 µM at the end of the experiment when the initial Ag-EN concentration was 1.85 and 9.27 µM, respectively.

As the particle surface area is an important factor determining the extent and kinetics of particle dissolution [17], the longer time for Ag^+^ to reach its maximum concentration in the two lowest nanoparticle concentration treatments manifests Ag^+^ release being limited by the total surface area of Ag-ENs under this condition. The Ag^+^ decrease after reaching the maximum may be because of the slow formation of insoluble Ag complexes with various ligands like chloride as described by Laban et al. [9]. Another possibility is the adsorption of Ag^+^ to the beaker walls. When Ag-EN dissolution is surface area limited, the Ag^+^ depleted in the media either through particulation or adsorption cannot be replenished right away and [Ag^+^]_T_ thus decreased with time. Besides the two possibilities above, it is also possible that Ag-EN solubility decreased as its specific surface area decreased with time for the two lowest nanoparticle concentration treatments [20].

When comparing the Ag-EN size distribution in the different nanoparticle concentration treatments at the same time point, no consistent trend was observed for the >35 nm fractions (35–200 nm and >200 nm fractions). However, significantly more Ag (p<0.05) were distributed in the <1 nm fraction while less Ag-ENs were distributed in the 1–35 nm fraction for the lower Ag-EN concentration treatments (Fig. 2). After 4 d mixing, 14.5%, 5.65%, 2.22%, and 1.18% of the total Ag-ENs were dissolved as Ag^+^ (<1 nm) while another 30.0%, 50.4%, 59.1%, and 59.8% were distributed in the 1–35 nm fraction when the initial Ag-EN concentration was 1.85, 9.27, 27.8, and 92.7 µM, respectively.

The observation that a higher percentage of Ag-ENs was dissolved at lower nanoparticle concentrations was also found by Laban et al. [9], which can be ascribed to the more substantial difference in Ag-EN concentration than that of [Ag^+^]_T_ between the different nanoparticle concentration treatments. Namely, the decrease of [Ag^+^]_T_ is less significant than the decrease of nanoparticle concentration itself. The lower [Ag^+^]_T_ in the lower nanoparticle concentration treatments was unexpected, as theoretically the Ag-EN solubility should be the same unless their nanoparticle size was different from each other [21], which was at least not observed based on the size fractionation results in the present study. Furthermore, there were still some Ag-ENs left, even in the lowest concentration treatments. Therefore, the different [Ag^+^]_T_ obtained in the different Ag-EN concentration treatments may also be explained by the surface-area-limited release of Ag^+^. Namely, the Ag-EN dissolution and possible Ag^+^ precipitation may be a dynamic process in the experimental system. The Ag^+^ depletion in the media could be quickly replenished in the two highest Ag-EN concentration treatments, which would be, however, not the case when its concentration is low.

### Effects of GSH

**Figure 3:**
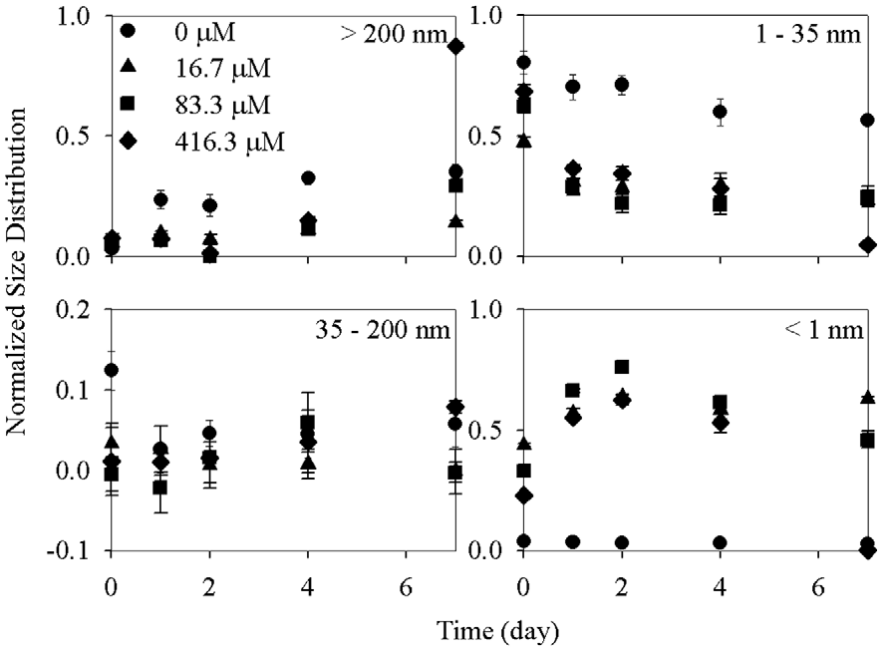
Normalized size distribution (to the initial Ag-EN concentration) of 27.8 µM Ag-ENs in different size fractions (>200 nm, 35–200 nm, 1–35 nm, <1 nm) during a 7 d period in the modified DY-V medium with the addition of 0, 16.7, 83.3, and 416.3 µM GSH, respectively. Data are mean ± standard deviation (n = 3).

When GSH was added, [Ag^+^]_T_ increased significantly (p<0.05) with time, and then plateaued or even decreased (Fig. 3). For example, [Ag^+^]_T_ increased from 12.1 µM at the beginning of this experiment to 17.7 µM on day 2 and then stayed constant at an ambient GSH concentration 16.7 µM. The other time-related trends (i.e., Ag-EN distribution in the 1–35 nm fraction decreased, while its concentration in the >200 nm and 35–200 nm fractions increased with time or kept relatively constant, respectively) were similar to those observed in the 20 d or nanoparticle concentration effect experiments described above and are not further described here. On the other hand, the presence of GSH significantly induced Ag-EN dissolution (p<0.05), with less Ag-ENs in the >200 nm and 1–35 nm fractions as compared with the control treatment. The only exception was that Ag concentrations increased abruptly in the particulate phase (>200 nm) at the end of the experiment especially when the GSH concentration was 416.3 µM. Accordingly, Ag concentration in the <1 nm and 1–35 nm fractions of this treatment decreased remarkably to 6.81 nM and 1.30 µM, respectively, on day 7. The increased Ag-EN dissolution in presence of GSH was also evidenced by a decreased Ag-EN light absorption (Fig. S2c), which can be taken as an indicator of Ag-EN dispersability.

Glutathione is a tripeptide composed of three amino acids, cysteine, glutamic acid and glycine. The sulphurhydryl group in cysteine is an extremely strong binding ligand (log K_a_ = 12.3) for the class B metal ion Ag^+^ [22]. The presence of GSH could thus induce the dissolution of Ag-ENs. It has been revealed that two mechanisms, 1) surface complexation and 2) solution coordination, are involved in chelating agent impacts on mineral (e.g., hematite and calcite) dissolution [23], [24], [25]. Glutathione could also be adsorbed onto the surface of Ag-ENs [12]. Therefore, surface complexation may be the major way through which Ag-ENs were solubilized when GSH was used. The longer time taken for [Ag^+^]_T_ to reach its maximum in the presence of GSH than in the control experiment may indicate Ag-EN solubilization was limited either by the adsorption of GSH to the Ag-EN surface or by the dissociation of GSH-Ag^+^ complexes from the nanoparticle surface. The second possibility is more likely as considering the rapidity of surface adsorption [26].

However, it was relatively unexpected that [Ag^+^]_T_ decreased consecutively after reaching its maximum when GSH was added, especially in the highest GSH concentration treatment. Furthermore, Ag-EN concentration in the 1–35 nm fraction also decreased abruptly at this high GSH concentration (Fig. 3). Together, these results imply that some Ag^+^ containing particulates were formed, especially at higher GSH concentrations besides the Ag-EN aggregates. Early work by Anderson [27] demonstrated that Ag^+^ could be strongly bound to GSH and the complexes thus formed have an aggregating nature. Such aggregates are in a layered structure by the stacking of a series of infinite columns composed of two chains of -Ag-S(R)-Ag-S(R)- zigzags [26]. Furthermore, GSH-Ag^+^ complexes are less stable than the cysteine-Ag^+^ complexes and tend to form gels in aqueous media [28]. Therefore, it is possible that the formation of GSH-Ag^+^ complexes in an aggregated form caused the decrease of Ag^+^, especially when the GSH concentration was high. Another possibility is that the GSH-Ag^+^ complexes are not stable and their organic fragment(s) may be oxidized leading to the production of mononuclear or polynuclear Ag-S complexes with lower solubility [29].

### Toxicity of Ag-ENs to the freshwater alga

**Figure 4:**
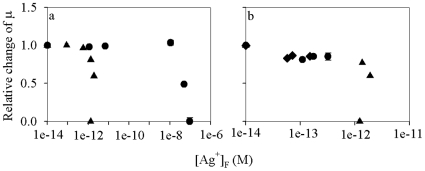
Relative changes of the cell-specific growth rate (µ) in the treatments with different free Ag^+^ concentrations ([Ag^+^]_F_, M) to that in the control in the experiment (a) where different concentrations of Ag-ENs (27.8, 92.7, 139.1, 185.4, and 278.1 µM; triangle symbol) and Ag^+^ (55.4, 74.2, 81.9, 83.4, and 92.7 µM; circle symbol) were added and (b) where different concentrations of GSH (triangle - 83.3 µM, circle - 249.8 µM, diamond - 416.3 µM) were applied for the four different Ag-EN concentration treatments (0, 139.1, 185.4, and 278.1 µM), respectively. Data are mean ± standard deviation (n = 3).

In the experiment, where the toxicity of Ag-ENs and Ag^+^ was compared, [Ag^+^]_T_ in the <1 nm fraction of each treatment was quantified and [Ag^+^]_F_ was then calculated. The free Ag^+^ concentrations were 0.088, 0.57, 1.34, 1.89, 1.31 pM for the five Ag-EN addition treatments (with nanoparticle concentrations from low to high), and 1.13 pM, 6.87 pM, 10.8 nM, 50.1 nM, 93.7 nM for the five Ag^+^ addition treatments. In order to see whether the potential toxicity of Ag-ENs could be well explained by the Ag^+^ they released, the toxicity results were presented as the change of [Ag^+^]_F_ (Fig. 4a). If similar inhibitive effects were observed at the same [Ag^+^]_F_ for both the Ag-EN and Ag^+^ addition treatments, then it can be concluded that the toxicity of Ag-ENs was caused by the Ag^+^ released. Significant toxicity (p<0.05) to the freshwater alga *O. danica* was observed in the higher Ag-EN concentration treatments. Cell growth was inhibited by 18.8%, 40.3%, and 100% when Ag-EN concentration was 139.1, 185.4, and 278.1 µM, respectively, while their [Ag^+^]_F_ was kept relatively constant (1.31–1.89 pM). In contrast, no significant toxicity (p>0.05) was observed in the Ag^+^ addition treatments until [Ag^+^]_F_ was higher than 10.8 nM. The cell growth was inhibited by 51.1% and 100% when [Ag^+^]_F_ was 50.1 and 93.7 nM, respectively. Therefore, the Ag-EN addition treatments appears to be more toxic than that of the Ag^+^ addition treatments based on their [Ag^+^]_F_. Accordingly, the calculated [Ag^+^]_F_ based EC50 was much lower for the Ag-EN addition treatments than that for the Ag^+^ addition treatments (1.27 pM vs. 49.1 nM). The Ag^+^ toxicity to freshwater algae has been examined in several studies, with the EC50 observed ranging from 12 to 930 nM, which are consistent with what was obtained in the present study [30], [31].

Metallic nanoparticle dissolution may affect the algal growth in three ways. First, nanoparticles dissolve in the bulk media, with the released metal ions diffusing to the algal surface and imposing toxic effects after internalization. This phenomenon was observed in our earlier study [12]. Second, nanoparticles within the diffusion layer of algal cells or attached to the cell surface may dissolve and thereby provide additional metal ions directly to the algae. This may be exactly what was found by Navarro et al. [7], in which Ag-ENs were found to be more toxic than AgNO_3_ based on the [Ag^+^]_F_ in the toxicity media. However, such toxic effects were reduced in the presence of cysteine (another strong Ag^+^ binding ligand) and were completely eliminated when the cysteine concentration increased to 500 nM. Third, nanoparticles could enter the cells directly and liberate metal ions once inside the cells. Therefore, a comparable [Ag^+^]_F_ based EC50 between the Ag-EN and Ag^+^ addition treatments can only be observed if the first possibility above is the case. Otherwise, Ag-ENs will appear more toxic, which is what we found in the present study.

To further test whether the toxicity of Ag-ENs we observed could be explained by either of the latter two mechanisms described above, the three highest Ag-EN concentrations in the first toxicty experiment were chosen together with the control treatment. In the meantime, three different concentrations of GSH were used to adjust [Ag^+^]_F_. The increased concentration of GSH was found to be able to reduce [Ag^+^]_F_ substantially in the media. The free Ag^+^ concentration ranged from 1.23 to 1.90 pM when the GSH concentration was 83.26 µM. As the GSH concentration increased to 249.8 and then to 416.3 µM, [Ag^+^]_F_ dropped to 0.11–0.32 pM and further to 0.058–0.15 pM. In the lowest GSH concentration treatments, the cell growth was inhibited by 22.5%, 39.8%, and 100% when the Ag-EN concentrations were 139.1, 185.4, and 278.1 µM, respectively, as is consistent with the first toxicity experiment. Although the toxic effects were significantly reduced at higher concentrations of GSH (p<0.05), there was still about 13.1–18.6% inhibition of cell growth for all the six Ag-EN addition treatments. If the Ag-EN toxicity observed in the present study was caused by either of the first two mechanisms described above, then no toxicity should be observed when the GSH concentration is so high (249.8 and 416.3 µM) to completely bind with Ag^+^, which was obviously not the case. Although GSH may be partly degraded either abiotically or by the algal cells, the effects of such processes should be negligible considering the high concentrations of GSH used and the relatively short duration of the toxicity tests. Therefore, the Ag-EN toxicity in the presence high concentrations of GSH is not because of the Ag^+^ release outside the cells (including those adsorbed on the cell surface).

**Figure 5:**
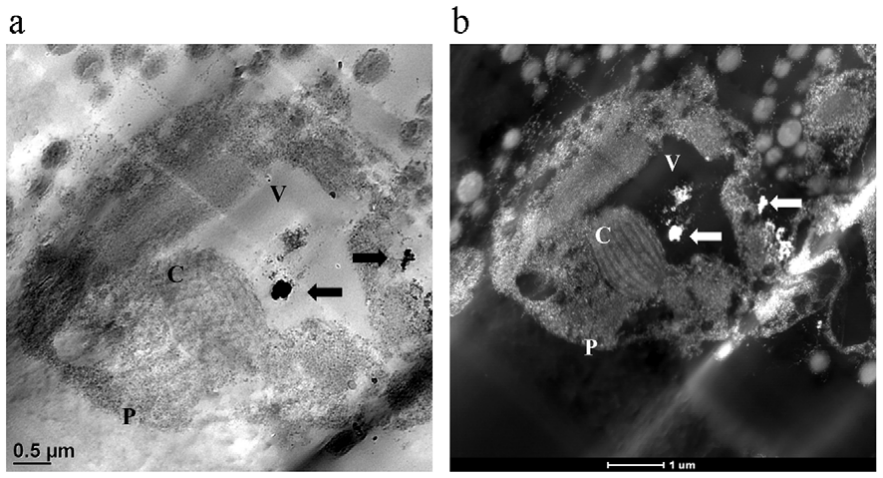
The transmission electron microscope (a) and Z-contrast dark-field scanning transmission electron microscope (b) images of a single *Ochromonas danica* cell in the Ag-EN addition (92.7 µM) treatment. Arrows indicates the locations of Ag-ENs inside the cells, which was further confirmed by energy dispersive X-ray spectrum. The letter ‘P’ represents the plasma membrane of the cell, ‘V’ means vacuole and ‘C’ is chloroplast.

In order to further find out whether there is any direct internalization of Ag-ENs into the cells, the alga in the control treatment as well as those from Ag-EN or Ag^+^ addition treatments were examined under the TEM and STEM. A noticeable amount of Ag-ENs was found in the vacuoles of *O. danica*, which was not observed in the control and Ag^+^ addition treatments (Fig. 5 and Fig. S3). The existence of Ag-ENs inside the vacuoles was further confirmed by the elemental composition profile obtained from an energy dispersive X-ray spectrometer shown in Fig. S4. The irregular shape of the vacuoles is possibly because of the distortion when preparing the samples for TEM analysis. There were no obvious toxic effects in all the treatments chosen for TEM analysis, excluding the possibility that it was an increase of cell membrane permeability or a break in the membrane that resulted in the passive uptake of Ag-ENs into the cells. Therefore, Ag-EN internalization into the cells was found in the present study to be an important mechanism through which algal growth was substantially reduced, especially in cells with endocytosis ability. However, it remains unclear whether Ag-ENs inside the cells can directly inhibit the algal growth or indirectly by the release of Ag^+^ internally.

The elimination of nanoparticle toxicity by different surface coatings was also observed in several other studies [32], [33], [34]. Possible explanations include the reduction of nanoparticle dissolution as well as the reduction of the direct interactions between nanoparticles and organisms. The first mechanism is less likely in the present study as the presence of GSH could significantly induce the release of Ag^+^ (p<0.05). For the second mechanism, the addition of GSH did not inhibit the attachment of Ag-ENs to the algal cell surface unlike what was observed by Li et al. [33], as part of the Ag-ENs already entered the cells. Therefore, the alleviation of Ag-EN toxicity under high GSH concentration in the present study may be due 1) to the inhibition of Ag-EN uptake into the cells by GSH, 2) to the reduction of the interaction between the internalized Ag-ENs and different organelles or 3) to the complexation of GSH with Ag^+^ inside the cells, all of which need to be further investigated. Significant toxicity from the intracellular Ag-ENs was observed at an extremely high nanoparticle concentration in the present study. Such effects may be attributed to the high GSH concentration used; however, Ag-ENs in the natural environment can potentially impose direct risk to the algae given the much lower ambient GSH concentration.

In conclusion, the surface functionalized Ag-ENs could be well dispersed in the experimental media under most conditions. However, they did not remain stable, and aggregates were continuously formed with time. In the meantime, a considerable amount of Ag^+^ was released by Ag-ENs with the final Ag^+^ concentration limited by the nanoparticle solubility. Glutathione, when added, could induce Ag-EN dissolution and lower [Ag^+^]_F_ concentrations. More importantly, remarkable uptake of Ag-ENs into the cells was found in the present study and these Ag-ENs inside the algal cells may contribute to the Ag-EN toxicity observed, even in the presence of GSH. To the best of our knowledge, this is the first report with direct evidence that nanoparticles could enter algal cells to exert their toxic effects.

## Supporting Information

Figure S1 Transmission electron microscope images of Ag-ENs in the (a) stock solution and (b) modified DY-V medium.
(TIF)

Figure S2 The UV-Visible light absorption spectrum of Ag-ENs (a) at the beginning (day 0) and end (day 20) of the 20 d experiment (Ag-EN concentration  =  27.8 µM); (b) at the end of the different Ag-EN addition experiment (Ag-EN concentration  =  1.85, 9.27, 27.8, 92.7 µM); and (c) at the end of the glutathione (GSH) addition experiment (GSH concentration  =  0, 16.7, 83.3 and 416.3 µM; Ag-EN concentration  =  27.8 µM).
(TIF)

Figure S3 The transmission electron microscope (a, c) and Z-contrast dark-field scanning transmission electron microscope (b, d) images of a single *Ochromonas danica* cell in the control (a, b) and Ag^+^ addition (c, d, 55.6 µM) treatments, respectively. Arrows in (c) and (d) indicates the locations where the energy dispersive X-ray spectrum was taken. The letter ‘P’ represents the plasma membrane of the cell, ‘V’ means vacuole and ‘C’ is chloroplast.
(TIF)

Figure S4 The representative energy dispersive X-ray spectrum of the arrow highlighted areas inside TEM or STEM images of the *Ochromonas danica* cell exposed either to 92.7 µM Ag-ENs (a) or to 55.6 µM Ag^+^ (b).
(TIF)

Text S1 Algal preparation for electron microscope.
(DOC)

Table S1 Compounds and their concentrations in the modified DY-V medium used in this study.
(DOC)

Table S2 Summary of experimental design for the three Ag-EN behavior and two toxicity experiments. DY-V medium without the addition of yeast extract was used for all the experiments.
(DOC)
