# Supplementary figures and images for: Intracellular Uptake: A Possible Mechanism for Silver Engineered Nanoparticle Toxicity to a Freshwater Alga Ochromonas danica
Source: PLoS One. 2010 Dec 22;5(12):e15196. doi: 10.1371/journal.pone.0015196 (PMC3008680; doi:10.1371/journal.pone.0015196)

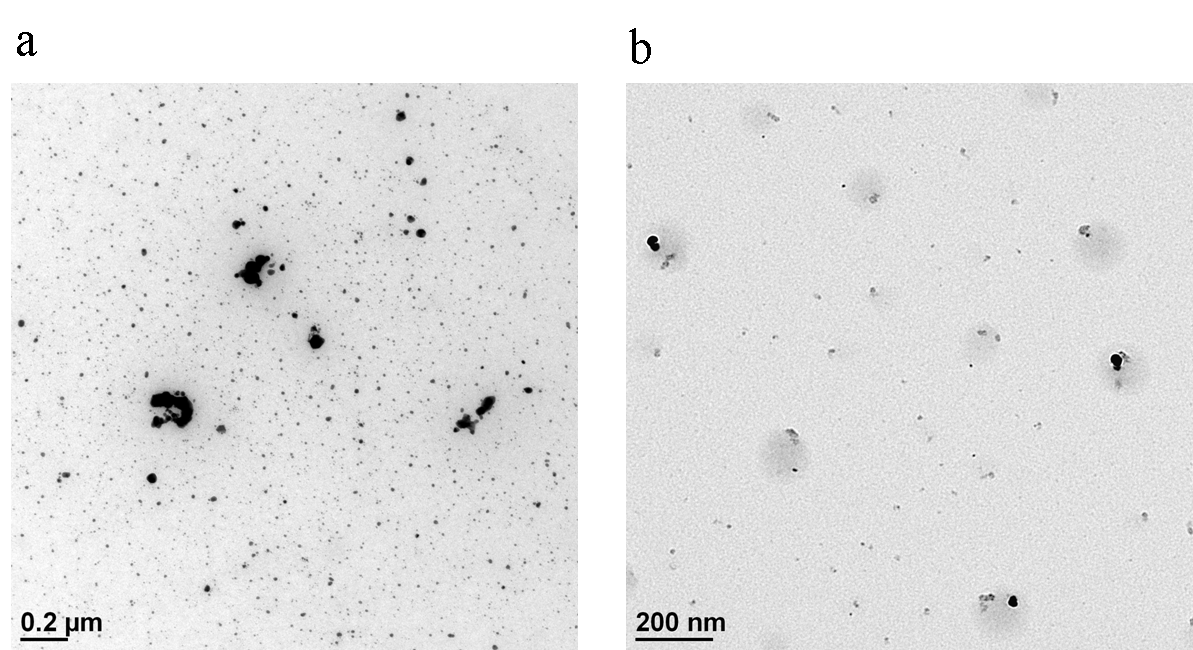

Supplement: Figure S1 — Transmission electron microscope images of Ag-ENs in the (a) stock solution and (b) modified DY-V medium. (TIF) [file pone.0015196.s001.tif]

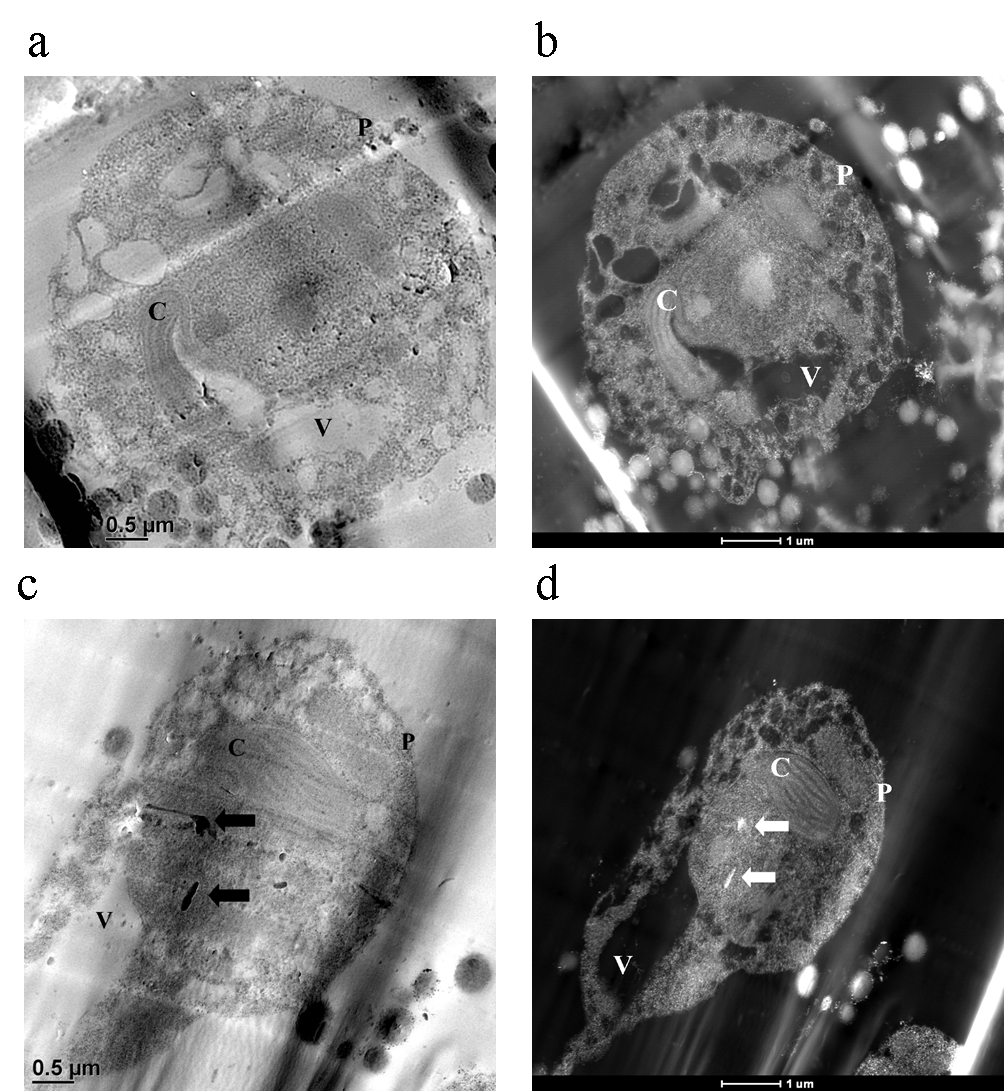

Supplement: Figure S3 — The transmission electron microscope (a, c) and Z-contrast dark-field scanning transmission electron microscope (b, d) images of a single Ochromonas danica cell in the control (a, b) and Ag+ addition (c, d, 55.6 µM) treatments, respectively. Arrows in (c) and (d) indicates the locations where the energy dispersive X-ray spectrum was taken. The letter ‘P’ represents the plasma membrane of the cell, ‘V’ means vacuole and ‘C’ is chloroplast. (TIF) [file pone.0015196.s003.tif]

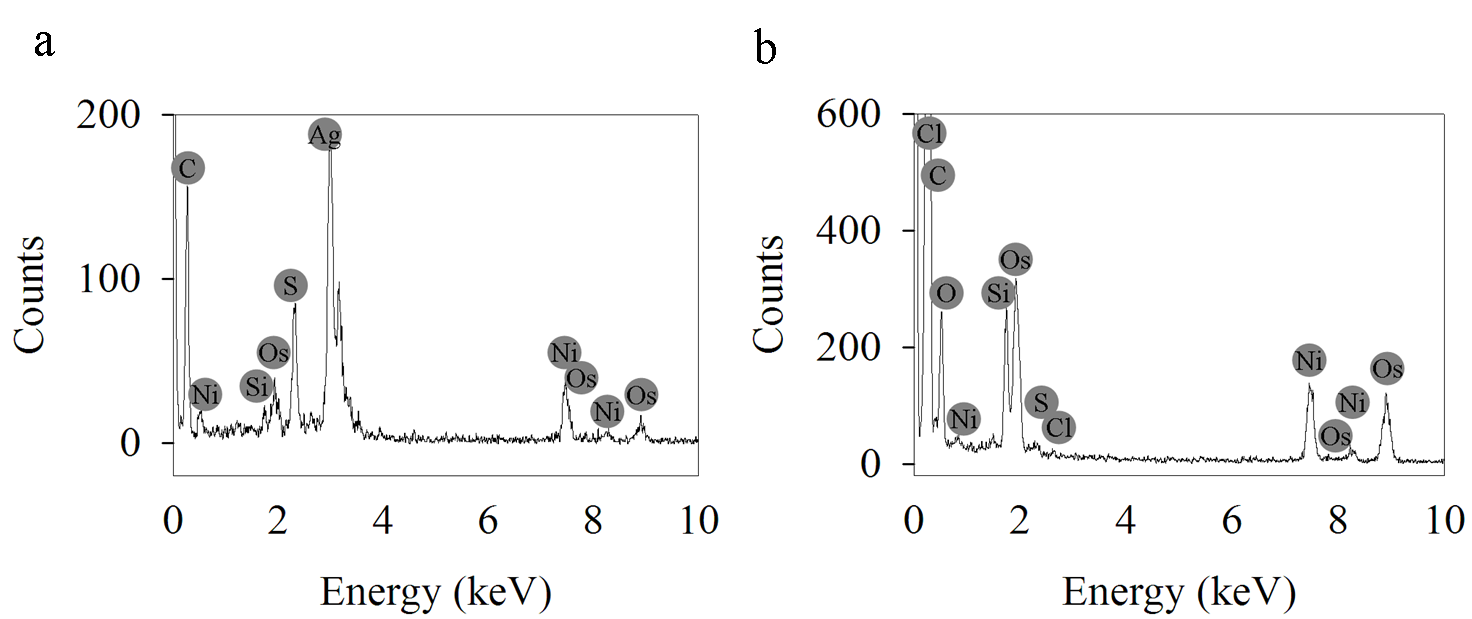

Supplement: Figure S4 — The representative energy dispersive X-ray spectrum of the arrow highlighted areas inside TEM or STEM images of the Ochromonas danica cell exposed either to 92.7 µM Ag-ENs (a) or to 55.6 µM Ag+ (b). (TIF) [file pone.0015196.s004.tif]
