# Supplementary material for: Intracellular Uptake: A Possible Mechanism for Silver Engineered Nanoparticle Toxicity to a Freshwater Alga Ochromonas danica
Source: PLoS One. 2010 Dec 22;5(12):e15196. doi: 10.1371/journal.pone.0015196 (PMC3008680; doi:10.1371/journal.pone.0015196)
Supplement: Text S1 — Algal preparation for electron microscope. (DOC) [file pone.0015196.s005.doc]

**Algal cell preparation for electron microscope**

In parallel to the first toxicity experiment of the present study, additional replicates were made in four treatments (control without any addition of Ag-ENs or Ag^+^, 27.8 and 92.7 μM Ag-ENs, 55.6 μM Ag^+^) to visualize how Ag-ENs may interact with the algal cells through transmission electron microscope (TEM) and Z-contrast dark-field scanning transmission electron microscope (STEM). The sample preparation was similar to what was decribed by Han et al. (Reference [15] in the main text). Briefly, the liquid algal samples from each treatment above were first mixed with Trump’s Fixative and then filtered through a 0.45 μm size plastic filter, so that the cells stayed on the surface of the filter. The cells were then stained in 1% (mg/ml, weight to volume ratio) osmium tetroxide (OsO_4_ in the solution of 0.1 M 4-(2-hydroxyethyl)-1- piperazineethanesulfonic acid buffer with pH 7.4) for about 0.5 h, and dehydrated through methanol (10-100%). Afterwards, they were embedded into epoxy resin and sectioned at 100 nm thickness to be analyzed by TEM and STEM. The elemental composition of the interesting areas of the TEM and STEM images was further analyzed by an energy dispersive X-ray spectrometer.
