# Supplementary material for: Intracellular Uptake: A Possible Mechanism for Silver Engineered Nanoparticle Toxicity to a Freshwater Alga Ochromonas danica
Source: PLoS One. 2010 Dec 22;5(12):e15196. doi: 10.1371/journal.pone.0015196 (PMC3008680; doi:10.1371/journal.pone.0015196)
Supplement: Table S1 — Compounds and their concentrations in the modified DY-V medium used in this study. (DOC) [file pone.0015196.s006.doc]

Table S1. Compounds and their concentrations in the modified DY-V medium used in this study.

| Compounds | Final Concentration |  | Compounds | Final Concentration |
| --- | --- | --- | --- | --- |
| 2-(*N*-morpholino)ethanesulfonic acid | 1.02 mM |  | Na_2_EDTA • 2H_2_O | 21.5 μM |
| MgSO_4_ • 7H_2_O | 203 μM |  | FeCl_3_ • 6 H_2_O | 3.70 μM |
| KCl | 40.2 μM |  | MnCl_2_ • 4H_2_O | 1.01 μM |
| NH_4_Cl | 50.1 μM |  | ZnSO_4_ • 7H_2_O | 139 nM |
| NaNO_3_ | 235 μM |  | CoCl_2_ • 6H_2_O | 33.6 nM |
| Na_2_ β-  glycerophosphate | 10.0 μM |  | Na_2_MoO_4_ • 6H_2_O | 82.7 nM |
| H_3_BO_3_ | 12.9 μM |  | Na_3_VO_4_ • 10H_2_O | 5.49 nM |
| Na_2_SiO_3_ • 9 H_2_O | 49.3 μM |  | H_2_SeO_3_ | 23.1 nM |
| CaCl_2_ • 2 H_2_O | 676 μM |  | thiamine · HCl | 296 nM |
| 3-(N-morpholino)propanesulfonic acid | 2 mM |  | biotin | 2.05 nM |
| Yeast extract | 0.5 g/l |  | cyanocobalamin | 0.369 nM |
| glucose | 1 mM |  |  |  |
