# Supplementary material for: Intracellular Uptake: A Possible Mechanism for Silver Engineered Nanoparticle Toxicity to a Freshwater Alga Ochromonas danica
Source: PLoS One. 2010 Dec 22;5(12):e15196. doi: 10.1371/journal.pone.0015196 (PMC3008680; doi:10.1371/journal.pone.0015196)
Supplement: Table S2 — Summary of experimental design for the three Ag-EN behavior and two toxicity experiments. DY-V medium without the addition of yeast extract was used for all the experiments. (DOC) [file pone.0015196.s007.doc]

Table S2. Summary of experimental design for the three Ag-EN behavior and two toxicity experiments. DY-V medium without the addition of yeast extract was used for all the experiments.

| Experiments | | Objective | Experimental design |
| --- | --- | --- | --- |
| Behavior Experiment | Mixing time effect | | **Ag-EN concentration:** 27.8 μM  **Experimental duration:** 20 d with seven time points (day 0, 2, 4, 7, 10, 15 and 20)  **Fractions of Ag quantified:** < 1 nm, < 35 nm, < 200 nm |
|  | Nanopartice concentration effect | | **Ag-EN concentration:** 1.85, 9.27, 27.8, 92.7 μM  **Experimental duration:** 7 d with five time points (day 0, 1, 2, 4, and 7)  **Fractions of Ag quantified:** < 1 nm, < 35 nm, < 200 nm |
|  | GSH effect | | **Ag-EN concentration:** 27.8 μM  **Experimental duration:** 7 d with five time points (day 0, 1, 2, 4, and 7)  **GSH concentration:** 0, 16.7, 83.3, and 416.3 μM  **Fractions of Ag quantified:** < 1 nm, < 35 nm, < 200 nm |
| Toxicity Experiment | Toxicity comparison of Ag-ENs with Ag^+^ | | **Concentration treatments:** totally 11 treatments with five Ag^+^ addition treatments (55.4, 74.2, 81.9, 83.4, and 92.7 μM) and five Ag-ENs treatments (27.8, 92.7, 139.1, 185.4, and 278.1 μM). The other one is control without any addition of Ag^+^ or Ag-ENs.  **GSH concentration:** 83.3 μM  **Experimental duration:** 2 d  **Toxicity endpoints:** cell specific growth rate  **Other parameters measured:** TEM and STEM images of the cells in certain treatments above. |
|  | GSH concentration effect | | **Concentration treatments:** totally 12 treatments with the matrix of four Ag-EN concentrations (0, 139.1, 185.4, and 278.1 μM) and three GSH concentrations (83.3, 249.8, and 416.3 μM).  **Experimental duration:** 2 d  **Toxicity endpoints:** cell specific growth rate |
